# Case report: A rare case of intragastric metastasis after liver transplantation for liver cancer

**DOI:** 10.3389/fonc.2024.1495517

**Published:** 2024-12-06

**Authors:** Youfeng Xue, Baojie Shi, Jianfa Zhong, Guimei Wang, Jie Wang, Wenbin An, Yunyun Qian, Zhaojie Su, Zhihai Peng, Hao Li

**Affiliations:** ^1^ Hepatobiliary Surgery Department, Fuqing Hospital, Fuzhou, Fujian, China; ^2^ Organ Transplantation Clinical Medical Center of Xiamen University, Department of Organ Transplantation, Xiang’an Hospital of Xiamen University, School of Medicine, Xiamen University, Xiamen, Fujian, China

**Keywords:** hepatocellular carcinoma, intragastric metastasis, liver cancer, liver transplantation, pediatric liver transplantation

## Abstract

A 13-year-old boy was admitted to Xiang’an Hospital of Xiamen University due to HBV-related liver cancer. Intrahepatic metastasis was considered to occur by CT scan. A gastroscope revealed esophagogastric variceal bleeding, and later, the patient underwent a successful liver transplantation. Fourteen months posttransplant, chest CT indicated lung metastasis, and the patient underwent thoracoscopic radical resection of lung cancer. Twenty-one months posttransplant, gastroscopy revealed a gastric fundus tumor growing into the gastric cavity. Proximal gastrectomy was performed, and pathology indicated moderately to poorly differentiated carcinoma without invasion of serosa, suggesting the first study to report HCC metastasis to the stomach lumen without invasion of serosa after LT. Currently, the alpha-fetoprotein (AFP) level of the patient has dropped below normal.

## Case report

In January 2021, a 13-year-old boy was enrolled in our center of liver transplantation at Xiang’an Hospital of Xiamen University because of HBV-related liver cancer.

**Figure 1:**
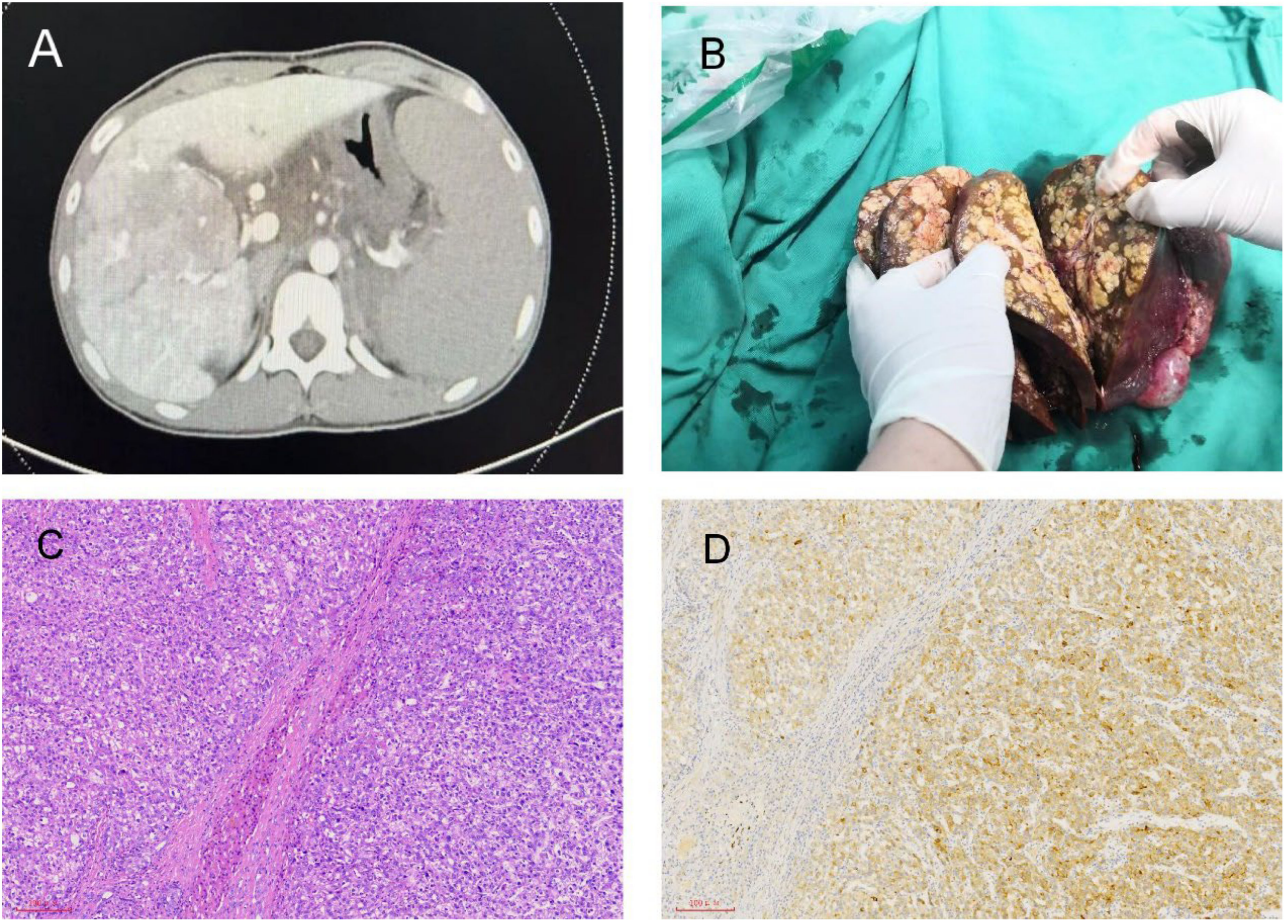
Radiological and pathological images of liver lesions. **(A)** Multiple intrahepatic lesions shown in CT. **(B)** Multiple nodules in the right lobe of the liver shown in the post-liver transplant specimen. **(C)** Liver H&E staining. **(D)** Liver immunohistochemistry: GPC3 (+).

The patient had been well until 2 weeks before this presentation, when diarrhea and bloody stool developed, which occurred 5-6 times/day and was accompanied by vomiting blood. After admission, a CT scan showed multiple intrahepatic masses (with the largest diameter of approximately 6.1cm, liver cancer with intrahepatic metastasis was considered), right portal vein cancer thrombus (not involving the main trunk of the portal vein, classified as Type I according to Chang’s classification), cirrhosis, splenomegaly, portal hypertension, and ascites (**Figure 1A**). Laboratory tests showed that AFP>1210 ng/ml, hemoglobin (HB) 98 g/L, and hepatitis B surface antigen were positive. Gastroscopy showed severe esophagogastric varices, and endoscopic variceal ligation was performed. The patient had been on oral antiviral therapy for HBV for more than ten years.

After 30 days of admission, the patient received hepatic arterial infusion of raltitrexed plus epirubicin. There was no evidence of extrahepatic metastasis on PET-CT. Four days prior to liver transplantation, the patient suddenly exhibited confusion and unresponsiveness to verbal stimuli. Vital signs at that time included a temperature of 36.3°C, a pulse rate of 142 beats per minute, blood pressure readings of 65/35 mmHg, a respiratory rate of 31 breaths per minute, and an oxygen saturation of 100% while on supplemental oxygen. Following rapid fluid resuscitation, volume expansion, and pressor support, the patient’s consciousness was regained, and his blood pressure stabilized at 110/62 mmHg. After 45 days of admission, the patient successfully underwent orthotopic whole liver transplantation (LT). Postoperative specimens revealed multiple nodules in the right lobe of the liver, with tumor diameters ranging from 0.3 cm to 4.5 cm (**Figure 1B**). Multiple tumors showed vascular invasion, and there was also a portal vein tumor thrombus. However, there was no hilar lymph node metastasis. The postoperative pathological examination revealed moderately differentiated hepatocellular carcinoma (**Figure 1C**). Immunohistochemistry showed that the expression of GPC3 was positive (**Figure 1D**). The patient received standard immunosuppressive drugs (tacrolimus 1.5mg twice daily), antiviral agents, and hepatic arterial infusion chemotherapy. The AFP level was reduced to 5.57 ng/ml, and PIVKA-II was decreased to 3.76 mAU/mL.

**Figure 2:**
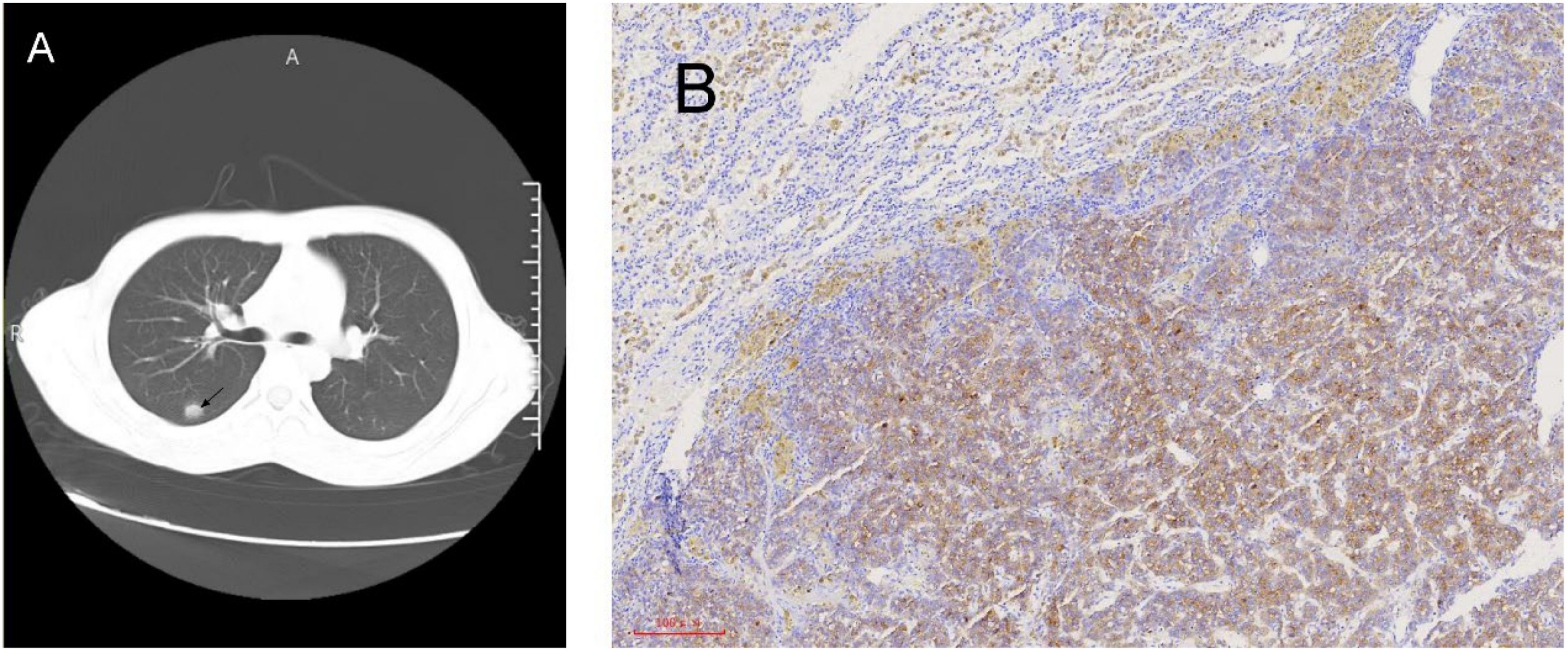
Radiological and pathological images of lung lesions. **(A)** Chest CT reveals right lung nodules. **(B)** Lung immunohistochemistry after thoracoscopic radical surgery for lung cancer: GPC3 (+).

In April 2022, the AFP level began to rise to 30 ng/ml. Chest CT detected new nodules in the right lungs, and lung metastases were considered (**Figure 2A**). After two months, the patient underwent thoracoscopic radical resection of lung cancer. The pathology showed a malignant tumor of the upper lobe nodule of the right lung, hepatocellular carcinoma-like differentiation (**Figure 2B**). Lenvatinib-targeted drugs were used, and AFP was regularly tested.

**Figure 3:**
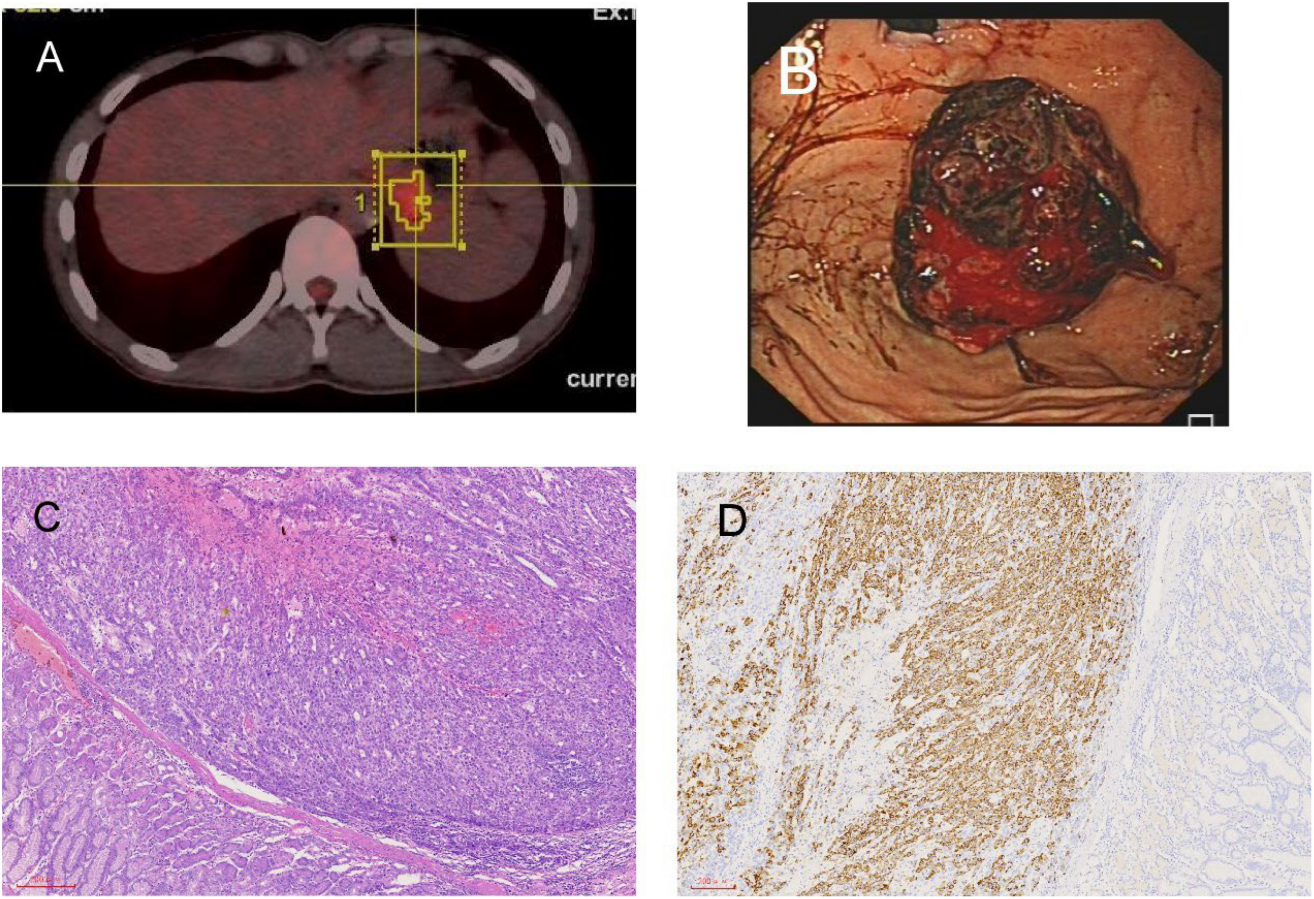
Radiological and pathological images of left adrenal gland and stomach lesions. **(A)** Newly detected tumor in the left adrenal gland shown in PET-CT. **(B)** The tumor grew from the fundus into the gastric cavity, and the gastric stromal tumor ruptured and bled. **(C)** Stomach H&E staining. **(D)** Stomach immunohistochemistry: hepatocyte (+).

In October 2022, the patient’s AFP level rose to 66.70 ng/ml. A PET-CT scan revealed multiple new masses, such as a mass in the left upper renal area (with a diameter of approximately 1.5cm), a mass in the left adrenal gland (measuring 1.3cm × 1.8cm), and a mass in the gastric cardia area (measuring 3.1cm × 2.3cm) (**Figure 3A**). The patient was given radiation therapy and continued Lenvatinib-targeted therapy. In January 2023, the patient underwent CyberKnife treatment for a left adrenal mass at an external hospital. After two months, the patient developed melena and hematemesis. Gastroscopy showed that the tumor was located in the fundus of the stomach and had grown into the lumen of the stomach, and stromal tumor rupture with bleeding was considered (**Figure 3B**). PET-CT showed that the mass of the fundus (4.1cm × 3.0cm) and the lateral side of the left upper renal pole (with a diameter of approximately 1.8cm) was larger than before, and the metabolism was increased. The left adrenal mass (1.3cm × 1.0cm) was smaller than before, and metabolism was reduced. The AFP level rose to 434 ng/ml. Due to repeated hematemesis and the fact that the bleeding could not be stopped by gastroscopy, the patient underwent radical gastric resection (proximal stomach) plus abdominal lymph node dissection. Pathology revealed a medium-poorly differentiated carcinoma. Immunohistochemistry showed Hepatocyte (+) (**Figures 3C, D**), in addition, GPC-3 and Arg-1 are also positive. Gastric metastasis of hepatocellular carcinoma was considered. Until now, the patient has been living and undergoing studies, and the AFP levels have returned to normal.

## Discussion

Liver cancer is one of the most common malignancies worldwide, ranking sixth (1–3). It is also the fourth leading cause of cancer-related deaths (1, 4). Hepatocellular carcinoma (HCC) is the most common form of liver cancer and accounts for nearly 90% of cases (5–7), and it stands as the third leading cause of cancer-related deaths worldwide (8). The incidence of HCC is increasing and is the main event leading to death in patients with cirrhosis (9). Intrahepatic metastasis is the most common metastasis of HCC, followed by lung metastases (10–12), and lung metastases are among the leading causes of hepatocellular liver cancer-related mortality (13). Liver cancer can directly invade the stomach but not intragastrically. Intragastric metastasis of HCC is very rare (14).

Several treatment modalities are available for patients with HCC, and LT remains the optimal treatment strategy for patients (15). The Milan criteria are the universally applicable standard of LT for HCC based on their significantly good outcome (16). HCC patients who meet the Milan criteria (single tumor <5 cm or up to three tumors <3 cm with no macrovascular invasion) are the most suitable for liver transplantation, with 5-year overall survival (OS) reaching 70% for HCC patients (16–18). For patients who were beyond the Milan criteria, the OS was no more than 3 months. After LT, the OS of these patients is more than 2 years. These results showed that liver transplantation can significantly promote the survival of patients who are beyond the Milan criteria. There was a prospective, randomized, controlled trial exploring the benefit of LT in patients who achieved successful and sustained downstaging of HCC exceeding the Milan criteria. These results showed that LT can significantly prolong patient survival beyond the Milan criteria. At the same time, the patient is relatively young, as HCC is uncommon in the pediatric population. The prognosis of HCC in children after liver transplantation (LT) differs from that of adult patients, with unique considerations for growth, development, and the long-term complications of immunosuppression. The one-year survival rate after pediatric liver transplantation is typically between 80% to 90%, and the five-year survival rate is between 70% to 80%. Although downstaging of HCC was not achieved in our patient after hepatic arterial infusion chemotherapy, in this study, LT still significantly increased the survival time of patients with HCC.

HCC frequently exhibits extrahepatic metastasis, with the typical sequence being the lungs, bones, lymph nodes, and adrenal glands (19). Extrahepatic recurrence typically develops after transplantation and almost always carries a better prognosis than intrahepatic recurrence (20). In our center of liver transplantation, approximately 60% of patients with HCC after LT experience extrahepatic metastases. For these metastatic patients, due to normal liver function, systemic chemotherapy, targeted therapy, or local radiation therapy is often given. Liver transplantation is the best option to remove liver cancer (21, 22). Postoperative recurrence is mainly due to hematogenous metastasis of circulating tumor cells (23, 24). This can explain the cause of lung and bone metastases after LT. Intragastric metastasis of HCC was very rare, and hematogenous metastasis, lymph node metastases, and local infiltration of tumors were not explained by the reasons for metastasis. To our knowledge, this is the first study to report HCC metastasis to the stomach lumen without invasion of serosa after LT. This patient had a portal vein cancer thrombus, high portal vein pressure and gastric esophageal varices leading to bleeding. The above reasons cause cancer cells to colonize the bleeding site through the portal vein to the left gastric vein and then through the fundusophageal vein. After liver transplantation, the low immunity of patients and acidic environment of the stomach both easily promote tumor cell growth.

In our study, we reported a rare metastatic pathway, intragastric metastasis of HCC. Liver transplantation can significantly prolong patient survival beyond the Milan criteria by downstaging.

## Data Availability

The original contributions presented in the study are included in the article/supplementary material. Further inquiries can be directed to the corresponding authors.
